# Motives for deliberate self-harm in a South African tertiary hospital

**DOI:** 10.4102/sajpsychiatry.v27i0.1524

**Published:** 2021-01-27

**Authors:** Petrus J.J. van Zyl, Jason Bantjes, Elsie Breet, Ian Lewis

**Affiliations:** 1 Department of Psychiatry and Mental Health, Faculty of Health Sciences, University of Cape Town, Cape Town, South Africa; 2 Institute for Life Course Health Research, Department of Global Health, Faculty of Medicine and Health Sciences, Stellenbosch University, Cape Town, South Africa

**Keywords:** deliberate self-harm, DSH, suicide, suicidal behaviour, self-injury, suicide, attempted

## Abstract

**Background:**

Although there is a growing body of literature on the epidemiology of deliberate self-harm (DSH) in South Africa, comparatively few studies have investigated the motives for self-harm. No studies have investigated the motives for DSH in Cape Town.

**Aim:**

The objective of the study was to identify the range of motives for DSH in Cape Town, and how these motives are associated with different socio-demographic factors, the severity of self-injury and levels of suicidal intent.

**Setting:**

Groote Schuur Hospital in Cape Town, South Africa.

**Methods:**

Data were collected from 238 consecutive patients presenting with DSH to the emergency department. The data were analysed by using bivariate and multivariate analyses.

**Results:**

Patients engaged in DSH for a range of motives. Interpersonal issues were the most common motive (70%), followed by financial concerns (22%). Male patients were twice as likely as female patients to report interpersonal motives for their self-harm. Patients who reported interpersonal issues were more likely to engage in methods of DSH that involved damage to body tissues. Patients without tertiary education were more likely to report academic concerns as a motive, and patients who reported psychiatric illness as motive for DSH were more likely to require medical interventions than those who did not.

**Conclusion:**

This study contributes novel insights into the motives for DSH in the Cape Town context and provides the foundation for continued research on the subject. The study also gives impetus to the development of therapeutic interventions focussed on the motives for self-harm.

## Introduction

Worldwide an estimated 804 000 people died from suicide in 2012, and this number is likely to continue rising.^1^ South Africa has an age-standardised suicide rate of 12.3/100 000, the 54th highest in the world.^1^ Suicide is a serious public health concern in South Africa.^2^ The development of locally appropriate suicide prevention programmes and guidelines for the treatment of suicidal patients is of great clinical importance. Deliberate self-harm (DSH) is both the strongest predictive^3^ and the most common^4,5^ risk factor found in those who have completed suicide. The risk of suicide in the year following an incident of DSH is 66 times that of the general population.^3^ Addressing DSH is therefore integral to suicide prevention. Various suicide risk assessment (SRA) tools are used in clinical practice to quantify suicide risk amongst patients presenting with DSH. However, these tools are of limited clinical utility as they generally produce high falsepositive rates and overestimate risk.^6,7^ This may be because the SRA tools do not adequately take account of patients’ motives for DSH.^8^

### Motives for deliberate self-harm

Although the terms *motive* and *intent* are often used as synonyms in suicide literature,^9^ it is clinically and theoretically useful to differentiate between these constructs. A motive is the underlying ‘cause or reason that […] induces action’.^10^ In contrast, intent describes the planned or desired outcome of the action taken. A motive is the psychological driver or *reason* to self-harm, whilst intent describes the desired *outcome* of DSH.

There are several challenges in determining the motives for DSH. Firstly, the patient’s explanation may not necessarily reflect the actual motive for his or her behaviour.^11^ Secondly, patients may feel the need to excuse or justify their behaviour.^12^ Thirdly, many different motives and intentions may be present concurrently.^13^

Finally, others may have views about the motives that are different from those of the patient.^13,14^ Despite these difficulties, researchers have often emphasised the importance of exploring motives for DSH in SRA.^14,15,16^

A number of studies have investigated DSH motives in South Africa. Most of these report suicide data in ways that make meaningful analysis and comparisons difficult^17^ and describe small, heterogeneous cohorts and many do not specifically address the issue of motives for DSH. None of these studies have specifically investigated Cape Town populations. The studies do, however, provide some insight into the local patterns of DSH in South Africa at large.

Mpiana and colleagues^18^ described a small cohort of eight patients who presented to Voortrekker Hospital (Limpopo Province, South Africa) following ‘parasuicide’. The authors found that economic and health-related factors as well as substance abuse and disturbed interpersonal relationships contributed to DSH, along with other factors. Du Toit and colleagues^19^ profiled 259 patients engaged in DSH who presented to Pelonomi Hospital (Free State Province, South Africa) over 1 year. The study found that problematic relationships (*n* = 143, 55.4%), financial problems (*n* = 59, 22.9%), psychiatric problems (*n* = 57, 22.1%), arguments (*n* = 51, 19.8%) and abuse (*n* = 47, 18.2%) were the most frequently cited ‘precipitants’. Obida and colleagues^20^ described 10 intentionally selected patients from Tshilidzini Hospital who engaged in DSH (Limpopo Province, South Africa). These patients cited unemployment, poverty, domestic violence, interpersonal conflicts, issues related to HIV, the death of the patient’s mother, depression, hopelessness and worthlessness and psychotic symptoms as motives for DSH. One participant cited accusations of witchcraft as contributing to the actions. Raubenheimer and Jenkins^21^ evaluated 39 patients engaged in DSH who presented to George Hospital (Western Cape, South Africa) during a 6-month period. Disagreement with a loved one was reported to be the main contributing factor in 21 (54%) of the participants, followed by stress at home (*n* = 13, 33%), financial worries (*n* = 6, 15%), intimate partner violence (*n* = 4, 10%) and psychiatric illness (*n* = 4, 10%). Ani and colleagues^22^ reviewed 215 patients engaged in DSH who presented to a KwaZulu-Natal emergency centre over 1 year. They found that relationship issues (*n* = 113, 53%) was the most reported motive, followed by ‘circumstance challenges’ (*n* = 64, 30%) and medical problems (*n* = 11, 5%). Because no studies have specifically investigated DSH motives in Cape Town, this is the first one to do so. We also build on the existing literature in this area by exploring the demographic and clinical factors associated with different motives. Detailed and accurate epidemiological data are the cornerstone of planning effective public health suicide prevention strategies, and therefore this study could aid in the development of appropriate suicide prevention planning for Cape Town.

## Methods

### Study design, setting and sampling

We set out to (1) document the range of motives for DSH in our cohort, (2) determine the socio-demographic correlates of different motives, (3) determine the associations of different motives with different levels of suicidal intent and (4) determine the associations of different motives with the severity of injuries.

Definitions of DSH are highly contested and the construct is difficult to operationalise.^23^ For this study, we defined DSH in accordance with the World Health Organization/EURO Multi-Centre Study on Parasuicide as:

An act with non-fatal outcome, in which an individual deliberately initiates a non-habitual behaviour that, without intervention from others, will cause self-harm, or deliberately ingests a substance in excess of the prescribed or generally recognised therapeutic dosage, and which is aimed at realising changes which the subject desired via the actual or expected physical consequences. (p. 74)^24^

The term, as used in this study, includes patients who engaged in self-harm with an intent to die as well as those with no intent to die.

For this study, a cross-sectional retrospective chart review was performed. Data were collected from 270 consecutive patients engaged in DSH who presented to the emergency department (ED) at Groote Schuur Hospital in Cape Town, South Africa, between 16 June 2014 and 29 March 2015. This sub-study analysed the data collected as part of a larger study titled *An investigation of the epidemiology, psychosocial correlates and cultural context of deliberate self-harm in South Africa.* The larger project is a joint study between the Department of Psychology at Stellenbosch University and the Department of Psychiatry and Mental Health at the University of Cape Town and has resulted in publications describing the methods of self-harm^25^ and associations between DSH and substance use.^26^ The data we present here have, however, not been previously reported.

Patients were clerked by medical staff in the ED as part of routine service delivery. Data pertinent to this study were then extracted from the clinical files and recorded on pro forma data collection forms by an experienced psychiatric nurse. Quality checks were conducted. After exclusion criteria were applied, 238 patients were included. Cases were excluded if their files were missing or insufficient information was available in the patient file (17 patients), if the patient had already been included in the sample on a prior presentation to the hospital during the period of data collection (9 patients), if the patient discharged himself or herself from hospital before data were captured (1 patient) or if the patient died as a result of his or her injuries (5 patients). A further 25 patients were excluded because they had missing data pertaining to the outcome variable (motive), and 3 patients were excluded because they reported their self-harm as being ‘a mistake’ rather than deliberate. The total number of participants in this sub-study is therefore 210.

### Measures

The following data were collected.

#### Demographic information

Each patient’s age, gender, relationship status, number of dependents, level of education and employment status were recorded. Socio-economic status (SES) was recorded as low SES (ZAR0 to ZAR76800) and high SES (ZAR76801 to ZAR2547601), based on annual family income.

#### Motives for deliberate self-harm

The patient’s ‘stated reasons’ for engaging in DSH were recorded and were taken to reflect his or her motive for DSH. The stated reasons were grouped into the following motives: ‘financial concerns’, ‘marital/romantic relationship issues’, ‘family conflict’, ‘medical illness’, ‘psychiatric illness’, ‘bereavement’, ‘academic concerns’, ‘unplanned pregnancy’, ‘not known’ and ‘other (specified)’.

#### Method of deliberate self-harm

Information relating to the method of DSH was captured. This included overdosing on ‘prescription’ or ‘non-prescription’ medication, the ‘ingestion or inhalation of poison’, the infliction of a ‘gun shot’ or ‘laceration’ as well as DSH by ‘immolation’, ‘hanging’ or ‘asphyxiation’.

#### The severity of deliberate self-harm

The severity of DSH was captured by using two variables: (1) whether a medical intervention was required (with options being ‘none’, ‘sutured’, ‘activated charcoal’, ‘oral medical treatment’, ‘IV medical treatment’, ‘intubation and ventilation’, ‘dialysis’ or ‘surgical treatment’) and (2) the patient’s Glasgow Coma Scale (GCS)^27^ score on admission to the ED. The GCS was used to measure the level of responsiveness to stimuli (i.e. the level of consciousness, [LOC]). For this study, we regarded a score of 13–15 to indicate no or minimal depression in the LOC, a score of 9–12 to indicate a moderately depressed LOC and a score of 8 or less to indicate a significantly depressed LOC.

#### Suicidal intent

Suicidal intent was measured in two ways: (1) the patients’ stated intentions were recorded, and this information was used to identify patients who said they intended ‘to die’ as a result of their injuries, and (2) the 12-item Pierce Suicidal Intent Scale (PSIS)^28^ was used to objectively measure the level of suicidal intent. We regarded a PSIS score of below 12 as ‘low to moderate suicidal intent’ and a score of 12 and above as ‘high suicidal intent’.

### Data analysis

Data were captured, cleaned and analysed by using version 19 of the Statistical Package for the Social Sciences (SPSS Inc., Chicago, IL, USA). Descriptive statistics were used to describe the sample characteristics and range and distribution of motives for DSH. Multiple correspondence analysis was used to investigate motives that were highly correlated with one another. This was represented in a correlation matrix and values below 0.30 were taken to indicate a weak correlation, whilst values of 0.30 and more indicated a moderate to strong correlation. Motives which had a moderate to strong correlation were merged into one representative motive. As such, ‘marital or romantic issues’ and ‘family concerns’ were merged into the motive ‘interpersonal issues’, whilst ‘isolation’, friendship problems and ‘legal problems’ were merged into the motive ‘social issues’. Univariate logistic regression analysis was used to explore the associations between socio-demographic factors, clinical characteristics and intentions associated with different motives. Then multivariate logistic regression analysis was used to estimate the best fit models to determine the factors that were associated with different motives for DSH, whilst controlling for socio-demographic variables. Results were reported as adjusted odds ratios (aORs) with 95% confidence intervals (95% CIs). For all statistical tests, the level of significance was set to *α* = 0.05.

Descriptive statistics were used to describe the socio-demographic and clinical characteristics of the sub-groups of patients who reported bereavement (*n* = 11) and unplanned pregnancy (*n* = 3) as motives for DSH, because both had sample sizes below that recommended for valid logistic regression analysis.^29^

### Ethical consideration

The data used in this study were collected as part of a larger study that received ethical approval from the Human Research Ethics Committee (HREC REF: 572/2019) of University of Cape Town as well as from the appropriate hospital authorities prior to data collection. This sub-study was granted additional ethical approval from the HREC. The information collected from each patient record was assigned a unique number and stored on a password-protected computer to protect patient confidentiality.

## Results

### Demographic characteristics of the sample

**FIGURE 1:**
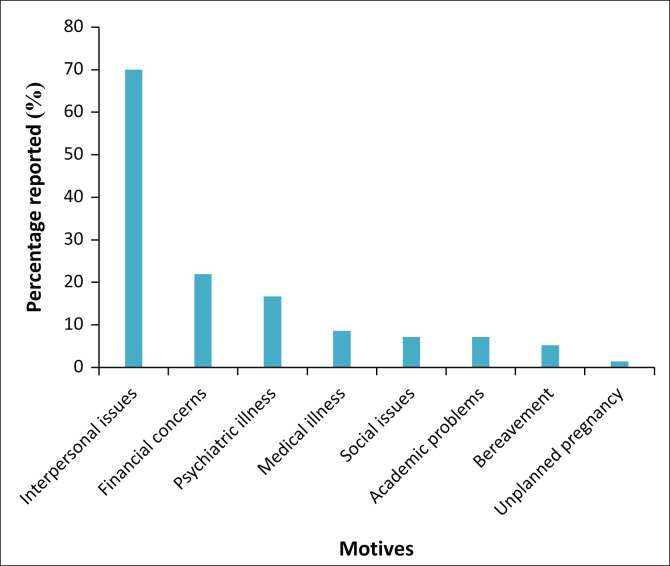
Percentage of reported motives for deliberate self-harm.

The mean age of the sample was 31.7 years (*SD* = 14.3, range: 18 to 82 years old). The majority of patients were female (*n* = 128; 61%), were not in a relationship (*n* = 167; 80%), had no dependents (*n* = 134; 64%), had no tertiary education (*n* = 176; 84%), were either unemployed or still studying (*n* = 161; 77%) and were of low SES (defined as earning less than R76 800 per annum; *n* = 118; 56%). In 37% of cases (*n* = 78), more than one motive was reported. As shown in Figure 1, more than two-thirds of patients reported interpersonal issues as the motive for DSH (70%).

### Socio-demographic factors associated with different motives for deliberate self-harm

Bivariate and multivariate regression analyses were used to explore associations between socio-demographic factors and motives for DSH. These results are summarised in Tables 1 and 2.

**TABLE 1:** Bivariate logistic regression analysis of socio-demographic variables associated with motives for deliberate self-harm (*n* = 210).

Variables	Predictor distribution in total sample (%)	Interpersonal issues as motive for DSH		Social issues as motive for DSH		Academic problems as motive for DSH		Financial concerns as motive for DSH		Medical illness as motive for DSH		Psychiatric illness as motive for DSH	
		OR	95% CI	OR	95% CI	OR	95% CI	OR	95% CI	OR	95% CI	OR	95% CI
Gender (male)	39.0	1.82	1–3.32*	0.96	0.33–2.80	1.83	0.56–5.97	0.996	0.51–1.95	1.01	0.37–2.71	0.47	0.23–0.99*
Relationship status (not in a relationship)	20.0	3.94	1.47–10.6*	0.5	0.13–2.73	–	–	0.53	0.21–1.35	–	–	0.62	0.22–1.70
Dependents (no dependents or pregnant)	63.8	0.58	0.30–1.11	0.8	0.27–2.36	3.81	0.84–17.4	3.81	0.84–17.4	1.34	0.45–3.96	2.05	0.88–4.78
Completed level of education (completed primary or secondary school)	83.8	0.75	0.35–1.62	0.35	0.05–2.76	5.44	1.83–16.2*	1.93	0.86–4.32	1.04	0.28–3.80	1.09	0.41–2.86
Employment status (unemployed)	76.7	1.24	0.59–2.60	0.87	0.23–3.21	0.52	0.11–2.38	1.37	0.64–2.93	0.68	0.19–2.46	1.05	0.44–2.49
SES (high SES, i.e. monthly family income ZAR76 801 to R76 800)	34.3	1.19	0.63–2.23	0.98	0.31–3.10	0.35	0.11–1.13	0.89	0.44–1.83	0.51	0.18–1.46	1.02	0.47–2.24

DSH, deliberate self-harm; OR, odds ratio; SES, socio-economic status; 95% CI, 95% confidence interval.

* , *p* < 0.05.

**TABLE 2:** Multivariate logistic regression analysis of socio-demographic factors as predictors of motives for deliberate self-harm as the outcome (*n* = 210).

Variables	Predictor distribution in total sample (%)	Interpersonal issues as motive for DSH		Social issues as motive for DSH		Academic problems as motive for DSH		Financial concerns as motive for DSH		Medical illness as motive for DSH		Psychiatric illness as motive for DSH	
		OR	95% CI	OR	95% CI	OR	95% CI	OR	95% CI	OR	95% CI	OR	95% CI
Gender (male)	39.0	2.07	1.06–4.04*	0.66	0.20–2.16	2.61	0.64–10.6	1.41	0.64–3.12	1.22	0.37–4.00	0.54	0.25–1.20
Relationship status (not in a relationship)	20.0	2.88	0.96–8.63	0.27	0.03–2.46	–	–	0.34	0.10–1.16	–	–	0.77	022–2.69
Dependents (no dependents or pregnant)	63.8	1.01	0.45–2.23	1.81	0.50–6.59	0.76	0.144–4.06	1.48	0.61–3.60	1.35	0.37–4.98	0.56	0.20–1.57
Completed level of education (completed primary or secondary school)	83.8	1.00	0.40–2.50	2.36	0.27–20.5	4.41	1.08–18.0*	2.71	1.04–7.09*	1.47	0.27–8.03	1.22	0.40–3.72
Employment status (unemployed)	76.7	1.31	0.56–3.07	0.7	0.14–3.51	0.25	0.03–2.17	1.29	0.52–3.21	0.31	0.38–2.55	1.17	0.44–3.11
SES (high SES, i.e. monthly family income ZAR76 801 to R76 800)	34.3	1.32	0.66–2.63	0.9	0.24–2.63	0.54	0.14–2.06	1.27	0.56–2.87	0.48	0.15–1.55	1.05	0.46–2.42

DSH, deliberate self-harm; aOR, adjusted odds ratio; SES, socio-economic status; 95% CI, 95% confidence interval.

* , *p* < 0.05.

#### Interpersonal issues

As shown in Table 1, the bivariate models demonstrated that being male (OR 1.82; 95% CI: 1–3.32) or not being in a relationship (OR 3.94; 95% CI: 1.47–10.6) was significantly associated with interpersonal motives for DSH. In multivariate models, being male (OR 2.07; 95% CI: 1.06–4.04) was the only socio-demographic variable significantly associated with interpersonal issues as a motive for DSH (Table 2).

#### Financial concerns

No statistically significant associations were found between socio-demographic variables and reporting financial concerns as a motive for DSH in the bivariate analysis (Table 1). However, the multivariate analysis demonstrated that having not completed a tertiary education increased the likelihood of reporting financial concerns (OR 2.71; 95% CI: 1.04–7.09) as a motive for DSH (Table 2).

#### Academic concerns

In the bivariate analysis, patients who reported having not completed tertiary education were 5.44 times more likely to report academic concerns (95% CI: 1.83–16.2) as a motive for DSH (Table 1). This relationship remained significant (OR 4.41; 95% CI: 1.08–18.0) in the multivariate models when controlling for the effects of other socio-demographic variables (Table 2).

#### Psychiatric illness

In the bivariate analysis, females (OR 2.11; 95% CI: 1.01–4.40) were at higher risk of reporting psychiatric illness as a motive for DSH (Table 1). This association was no longer statistically significant (OR 1.85; 95% CI: 0.84–4.08) when controlling for other socio-demographic factors in the multivariate analysis (Table 2).

#### Social issues

In both the bivariate and multivariate analyses, no statistically significant associations were found between any socio-demographic variables and the reporting of social issues as the motive for DSH (Tables 1 and 2).

#### Medical illness

Reporting a medical illness as a motive for DSH was not associated with any of the socio-demographic variables we collected, in both bivariate and multivariate analyses (Tables 1 and 2).

#### Bereavement

Amongst patients who reported bereavement as a motive for DSH (*n* = 11), the majority were female (*n* = 7), were not in a relationship (*n* = 10), did not have any dependents (*n* = 7), did not have a tertiary education (*n* = 4), were unemployed (*n* = 7) and were of low SES (*n* = 6).

#### Unplanned pregnancy

Amongst patients who reported unplanned pregnancy as the motive for DSH (*n* = 3), all patients were not in a relationship, did not have a tertiary education, were unemployed and were of low SES. Of these patients, two reported that they did not have any dependents.

### Clinical factors associated with different motives for deliberate self-harm

This study investigated associations between patients’ stated motives for DSH and (1) method of DSH, (2) severity of injuries (i.e. LOC on admission and whether medical or surgical intervention was required) and (3) suicidal intent (i.e. score on the PSIS and expressing a wish to die). These associations were explored in bivariate logistic models (Table 3) and in multivariate models, controlling for socio-demographic variables (see supplementary material, Tables S1–S6).

**TABLE 3:** Bivariate logistic regression results of associations between motives for deliberate self-harm and method of self-harm, severity of injuries and suicidal intent (*n* = 210).

Variables	Method of deliberate self-harm				Severity of injuries				Suicidal intent				Impulsive act	
	Self-poisoning		Damage to body tissue		Depressed level of consciousness on admission as measured by GCS		Required a medical intervention		Pierce Suicide Intent Scale		Expressed wish to die			
	OR	95% CI	OR	95% CI	OR	95% CI	OR	95% CI	OR	95% CI	OR	95% CI	OR	95% CI
Interpersonal issues as motive for DSH	0.48	0.236–0.992*	2.71	1.23–5.96*	0.641	0.261–1.58	0.59	0.32–1.09	0.82	0.32–2.09	7.5	1.47–38.3*	1.71	0.257–11.4
Social issues as motive for DSH	0.649	0.140–3.00	2.48	0.313–19.6	2.55	0.323–20.1	1.74	0.60–5.00	–	–	–	–	–	–
Academic problems as motive for DSH	1.09	0.292–4.07	2.48	0.313–19.6	–	–	2.32	0.81–6.68	0.9	0.25–3.28	0.5	0.06–4.35	0.038	0.002–0.936*
Financial concerns as motive for DSH	0.89	0.378–2.010	1.16	0.443–3.03	0.775	0.32–1.87	0.47	0.22–1.02	0.31	0.13–0.75*	2.09	0.25–17.4	8.45	0.531–134.4
Medical illness as motive for DSH	1.27	0.39–4.08	0.828	0.225–3.05	1.42	0.311–6.52	0.95	0.4–2.64	1.04	0.25–4.41	0.043	0.01–0.198*	0.014	0.001–0.198*
Psychiatric illness as motive for DSH	3.34	1.50–7.45*	0.223	0.095–0.522*	1.05	0.372–2.95	2.35	1.13–4.92*	0.9	0.25–3.28	0.61	0.117–3.14	0.152	0.011–2.05

DSH, deliberate self-harm; OR, odds ratio; 95% CI, 95% confidence interval.

* , *p* < 0.05.

### Motives associated with methods of deliberate self-harm

In the bivariate analysis, patients who reported interpersonal issues were approximately 2.7 times more likely to use damage to body tissue as a method of DSH (95% CI: 1.23–5.96; Table 3). Those who reported psychiatric illness were less likely to report damage to body tissue as the method of DSH (OR 0.22, 95% CI: 0.10–0.52; Table 3). Both these associations endured in multivariate analysis (Tables S1 and S6). Additionally, in the multivariate analysis, males were at increased likelihood of engaging in damage to body tissue across various motives whilst controlling for other socio-demographic factors (Tables S1–S5). By contrast, in the bivariate analysis, patients who reported interpersonal issues as a motive were at lower risk of reporting self-poisoning as a method (OR 0.48; 95% CI: 0.24–0.99), whilst those who reported psychiatric illness were at increased risk of reporting this method (OR 3.34; 95% CI: 1.50–7.45; Table 3). In the multivariate analysis, the association between reporting interpersonal issues and not using self-poisoning as a DSH method remained significant (OR 0.343; 95% CI: 0.14–0.82; Table S1). The finding that psychiatric illness as a motive was associated with using self-poisoning also persisted during multivariate analysis (OR 4.21; 95% CI: 1.67–10.60; Table S6).

Of the 11 patients who reported bereavement as their motive, 8 reported self-poisoning as their method of DSH. All three patients who reported unplanned pregnancy as their motive for DSH used self-poisoning as the method for their DSH.

### Motives associated with the severity of deliberate self-harm

#### Level of consciousness on admission

Bivariate analysis showed no statistically significant associations between the motives for DSH and GCS scores on admission (Table 3). In multivariate models, having no dependents was a risk factor for moderate to significant depression in GCS when controlling for socio-demographic factors and various motives, whether interpersonal issues (OR 4.04; 95% CI: 1.11–14.70), social issues (OR 3.95; 95% CI: 1.08–14.40), academic problems (OR 4.15; 95% CI: 1.14–15.10), financial concerns (OR 4.27; 95% CI: 1.16–15.8) and medical illness (OR 4.09; 95% CI: 1.13–14.90; Tables S1–S5). All patients who reported bereavement and unplanned pregnancy as the motives for DSH received scores of minimal depression in LOC.

#### Requiring medical intervention

Patients who reported psychiatric illness as a motive for DSH were approximately 2.4 times more likely to require medical intervention (95% CI: 1.13–4.92; Table 3). This association remained significant (OR 2.44; 95% CI: 1.08–5.47) in the multivariate models, controlling for socio-demographic factors (Table S6). Just over half (*n* = 6; 54.5%) of patients who reported bereavement as their motive for DSH received a medical intervention. Two of the three patients who reported unplanned pregnancy as their motive for DSH received a medical intervention.

### Motives associated with suicidal intent

#### Level of suicidal intent

In the bivariate analysis, patients who reported financial concerns as a motive for DSH were less likely to score ‘high suicidal intent’ on the PSIS (OR 0.31; 95% CI: 0.13–0.75; Table 3). This association persisted during multivariate analysis (OR 0.34; 95% CI: 0.12–0.95), when controlling for socio-demographic factors (Table S4). Only two (18.2%) patients who reported bereavement as the motive for their DSH also received scores indicative of high suicidal intent. None of the patients who reported unplanned pregnancy as their motive for DSH received an assessment by using the PSIS.

#### Expressed a wish to die

In the bivariate analysis, patients who reported interpersonal issues as their motive were 7.5 times more likely to report a wish to die (95% CI: 1.47–38.3) than those who did not, whilst patients who reported medical illness as their motive were significantly less likely to report a wish to die (OR 0.04; 95% CI: 0.01–0.2; Table 3).

In the multivariate analyses, the association between interpersonal issues as a motive (Table S1) and medical illness as a motive (Table S5) was no longer significant, whilst controlling for socio-demographic factors. However, reporting academic problems as the motive for DSH was associated with decreased risk of expressing a wish to die (OR 0.14; 95% CI: 0.32–0.59), whilst controlling for socio-demographic factors (Table S3). Most (*n* = 8; 72.7%) of the patients who reported bereavement as their motive for DSH selected ‘to die’ as the intention for DSH. All patients who reported ‘unplanned pregnancy’ as their motive for DSH reported that they did not wish to die when they engaged in self-harm.

## Discussion

This study generated novel data about the range of motives for DSH that are reported by patients in a Cape Town ED, and the socio-demographic and clinical correlates of these motives. The study found that whilst patients engage in DSH for a variety of reasons, interpersonal issues are by far the most commonly cited motive, occurring in 70.0% of cases. This prominence of interpersonal issues as a motive for DSH is consistent with studies in other parts of the world.^30,31,32,33^ The finding is also consistent with contemporary theories of suicide, such as the interpersonal–psychological theory of suicidal behaviour.^34^ This is clinically significant as it highlights the need for clinicians to explore interpersonal factors when evaluating suicide risk in patients engaged in self-harm and to make use of interventions that explicitly address the underlying interpersonal conflict as a motive for self-harm. Several psychotherapeutic interventions aimed at reducing interpersonal conflict have proved effective in reducing the risk of future self-harm, including cognitive therapy, dialectical behaviour therapy, problem-solving therapy and interpersonal psychotherapy.^35^ The need to scale up psychotherapeutic services in the South African public health sector has become an important discussion point^36^ and is given further impetus by our findings.

Another noteworthy finding is that there is no single motive for DSH common to all patients. This finding is also consistent with international literature^8,14^ as well as with the results from other South African studies.^18,19,20,21,22^ The finding suggests that clinicians should be aware of the uniqueness of each patient’s experience and allow for narratives to fully unfold in each case. This also means that therapy should be flexible to address various possible motives as well as focus on ‘real-life’ stressors related to the home, academic and work environments.

Male patients engaged in DSH were twice as likely as females to report interpersonal reasons as a motive for DSH even when controlling for other socio-demographic factors. A possible explanation is that this finding reflects aspects of hegemonic masculinity, which remains prevalent in South Africa.^37^ Hegemonic models of masculinity typically entail values of competitiveness, emotional stoicism and self-reliance, whilst discouraging the expression of grief, sadness, anxiety and fear.^38^ Men who conform to these standards of hegemonic masculinity typically have trouble resolving interpersonal difficulties and accessing interpersonal support. They also tend to exhibit more externalising symptoms than men who do not conform to these standards.^36,37,38^ As a result, these men may battle to successfully negotiate interpersonal relationships and instead resort to self-directed violence. This area warrants further qualitative investigation.

Furthermore, the findings show that those who reported interpersonal reasons as a motive for DSH were almost three times more likely than other patients engaged in DSH to employ methods of self-harm that resulted in damage to body tissue, even when controlling for socio-demographic variables. This further highlights the centrality of interpersonal factors in the aetiology of self-harm and the need for this to be addressed in assessment and interventions with this patient group.

Finally, financial distress in various forms is a well-recognised risk factor for suicide.^39^ This appears particularly true with regard to the loss of prior financial status.^40^ It is interesting to note that in our study, those who reported financial distress as a motive for DSH had lower levels of suicidal intent. One possible explanation for this unexpected finding may be that the individuals in our study may have had long-standing financial distress rather than a loss in financial status.

## Limitations

This study is limited by several factors. Firstly, the study took place in a single Cape Town tertiary hospital. Secondly, the study has a relatively small sample size. Both these factors may limit the generalisability of the findings to a broader South African context. Thirdly, the study relies on self-reports of patients and the clinical judgement of ED medical officers, which may or may not reflect the patient’s true underlying narrative. Fourthly, this initial exploratory study used crude measures of severity. Subsequent work may focus on more subtle and meaningful variables of severity and to what extent these variables correlate with potential fatality. Fifthly, because data were collected by non-psychiatric staff, it is likely that psychiatric comorbidity in the cohort is either under- or overestimated. Sixthly, data on socio-cultural variables were not considered because these variables cannot be easily quantified for statistical analysis. It would, however, be helpful if future qualitative studies explored socio-cultural factors that may influence motives for DSH. Finally, resource restraints limit the ability to follow up patients, prohibiting a longitudinal analysis of long-term outcomes associated with different motives for DSH in our study.

## Conclusion

Deliberate self-harm is increasingly recognised as both a common and an important clinical entity that is a key predictor of completed suicide. As a result, research on DSH has increased, particularly over the past decade. Increasingly, the focus has been on the motives behind this behaviour to understand how best to reduce DSH and suicide. This quantitative study of patients in Cape Town who engaged in DSH contributes towards building a body of knowledge on the topic of motives for DSH in South Africa and lays a foundation for future research. The questionnaire used in this study (available from the authors) could be used in other hospitals or clinical settings to compare the motives for DSH in different populations. The tentative findings that have emerged from this study could be used as a starting point for qualitative studies, which could deepen our understanding of patients’ narratives around DSH.
